# The Effect of Abusive Supervision on Employees’ Work Procrastination Behavior

**DOI:** 10.3389/fpsyg.2021.596704

**Published:** 2021-01-28

**Authors:** Qi He, Mengyun Wu, Wenhao Wu, Jingtao Fu

**Affiliations:** ^1^School of Finance and Economics, Jiangsu University, Zhenjiang, China; ^2^Overseas Education College, Jiangsu University, Zhenjiang, China; ^3^School of Mangement, Hainan University, Haikou, China

**Keywords:** abusive supervision, workplace ostracism, work procrastination behavior, psychological resilience, psychological detachment

## Abstract

Work procrastination is a retreat behavior associated with negative cognitive experience and it results in great losses to individual as well as organizational development. Understanding the antecedents of employees’ work procrastination behavior contributes to lower frequency of its occurrence. This research builds a dual-moderated mediation model from the perspective of cognitive appraisal theory and explored work procrastination behavior of employees subjected to abusive supervision. With 378 valid returned questionnaires, data collected from 32 companies in Beijing, Shanghai, Tianjin, and Chongqing supports our hypotheses. This result has enriched the understanding of work procrastination behavior and provided practical implications to avoide its negative effects.

## Introduction

Procrastination is often dubbed the disease of era, with a prevalence rate in general population of about 20–25% (Ferrari et al., 2007). Office workers procrastinate about 1.3 h a day, and this number probably understates the truth (D’Abate and Eddy, 2007). Work procrastination behavior refers to the deliberate postponement of work tasks that are expected to be accomplished (Solomon and Rothblum, 1984), exerting profound and extensive effects on employees’ lives and organizational development (Bolden and Fillauer, 2019). It is vital to explore the antecedents of work procrastination behavior as it provides theoretical explanations and practical guidance on how to effectively avoid these problems. Previous research claimed that individuals’ irrational expectations and perceptions of target motivation (Ainslie, 1975), workload (Folkman, 1984), or task nature (O’Donoghue and Rabin, 1999) are accompanied by negative emotional experience, and trigger work procrastination behavior. Those researches regarded work procrastination behavior as an active choice after employees’ evaluation of target task and decision to delay task completion in order to allocate more attention for other things. However, there could also be passive choices under workplace pressures, especially among the employees in Chinese organizations.

Cognitive appraisal theory explains the effect of environment on behaviors from cognitive perspective (Lazarus, 1966). Abusive supervision begets employees’ psychological distress, then throw them into negative thoughts and heavy feelings (Peltokorpi and Ramaswami, 2019). Employees may fall into self-deprecation, and feel low job competency and lack of self-worth. Inevitably their ability to interact equally with other members will be undermined (Yang et al., 2020). As a consequence, workplace ostracism in the cognitive structure will take place with a psychological cognitive state formed by individuals’ objective perception and understanding of work situation. Workplace ostracism will reduce information communication and job feedback, further cause difficulties for employees to obtain job opportunities and accumulate work experience. It becomes challenging to meet emotional needs to maintain connections and build identity for employees, and likely to damage employees’ trust and recognition to the organization (Richard et al., 2020). Thus employees’ work motivation will be impaired. In summary, a comprehensive exploration of the antecedent of work procrastination behavior should consider: (a) How abusive supervision causes workplace ostracism; (b) How workplace ostracism affects work procrastination behavior.

Individual’s perceptual response to environmental events is a complex conceptual evaluation process (Lazarus, 1991). Meanwhile psychological resilience activates different adaptations to external environment (Patryk, 2019) and result in differences among cognitive processes and evaluation outcomes. Psychological resilience empowers employees to overcome difficulties (Anasori et al., 2020) by adapting to environmental changes, then manage self-cognition, buffer cognitive deviation induced by abusive supervision, and reduce negative evaluations of organizational environment. Also, different processing methods for cognitive evaluation will result in different emotional and behavioral outcomes (Lazarus, 1966). The recovery process of psychological detachment will increase employees’ positive emotions (Fritz et al., 2010) and affect their behavioral choices (Marcus and Roy, 2019). If employees can restore psychological capital away from work situations, they will have more coping resources to alleviate the negative cognitive and emotional pains (Park et al., 2018). However, individuals with low psychological detachment are difficult to continuously invest emotional resources in the face of workplace ostracism and they tend to alleviate interpersonal pressure and the sense of exhaustion by postponing task process.

**FIGURE 1:**
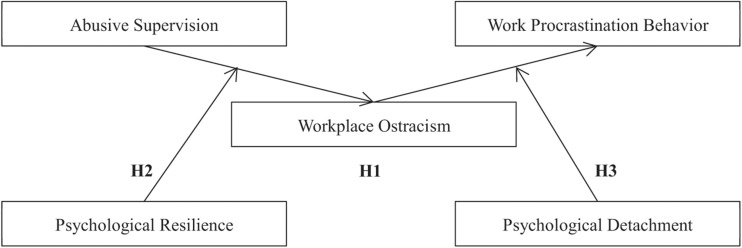
Theoretical model.

Hence, this research explored the effect of abusive supervision on employees’ work procrastination behavior based on cognitive appraisal theory. Workplace ostracism is a possible perception of employees who encounter abusive supervision, and can predict the occurrence of employees’ work procrastination behavior. We also anticipated that psychological resilience and psychological detachment will moderate this process. The theoretical model can be seen in Figure 1.

## Theoretical Basis and Research Hypothesis

### Cognitive Appraisal Theory

Lazarus (1966) proposed cognitive evaluation between environmental stimuli and emotional response, described the emotional process of situational evaluation and subsequent behaviors around cognitive structure (Lazarus and Cohen, 1987). According to cognitive appraisal theory, employees conduct cognitive processing and meaning construction based on informational clues obtained from organizational context, and adjust behaviors in accordance with cognition. Emotion is part of individuals’ perception of environmental information and stimulus events, and triggers behavior changes (Lazarus, 1991). Therefore, environmental information, cognitive evaluation, and behavioral results jointly determine individuals’ emotions, as well as describe the process from perception to action. Lazarus (1966) advocated that the development of emotions depends on individual’s perception of environmental information, and determines their measures through cognitive evaluation, which leads to two core concepts of “appraisal” and “coping” for in-depth analysis (Lazarus, 1984).

“Appraisal” refers to individuals’ information evaluation of environmental stimuli. Individuals need to process information on situational events (Lazarus, 1991) and develop a subjective interpretation of the objective situation. Cognition and evaluation of environmental information are also regulated by psychological traits. Degree of response adjustment and control is involved and it produces different meaning judgments and pressure perceptions. This difference further leads to cognitive discrepancies. “Coping” is an action promoted by individuals’ cognitions. Individuals will activate emotional system based on the cognitive assessment, which is intended to effectively alleviate the negative emotions caused by cognitive process through emotion-centric response measures (Lazarus, 1991). The process of behavioral response prediction also emphasizes that coping resources are the main factor that restricts emotional experience with involvement of individuals’ attempts to change the emotional state (Lazarus, 1982), thus produce different feedback results.

Therefore, the basic assumption of cognitive appraisal theory is environmental and behavioral components jointly determine the theoretical theme, accompanied by individuals’ cognition and evaluation of stress events, also recognizing the moderating effect of personality traits.

### Abusive Supervision, Workplace Ostracism, and Employees’ Work Procrastination Behavior

#### Abusive Supervision and Workplace Ostracism

Workplace ostracism is defined as employees’ perception when they are ignored, rejected, o treated differently by other organizational members in the workplace (Ferris et al., 2008). As a psychological feeling, workplace ostracism largely depends on the subjective criteria for judging and assessing others’ attitudes or behaviors, entailing a negative interpersonal violence. Therefore, workplace ostracism is not only a response of stressful situation, but also employees’ mental discomfort. Employees cognize self-concept and self-worth based on relevant information conveyed by others, then form a subjective cognition of their value and status (Nolan and Rowley, 2020). Interpersonal setbacks and tension may easily destroy emotional communication within the interpersonal network and lead to negative subconscious, seriously jeopardizing individuals’ sense of environmental control and belonging. Thus subjective alienation will ensue, such as interpersonal panic and workplace loneliness.

Abusive supervision is defined as leaders’ continuous verbal and non-verbal aggression (Tepper et al., 2006), mainly emphasizing a low-quality interpersonal dissonance and friction. It usually conveys emotional destruction information such as contempt and disgust. As a result, individuals hold a suspicious, even negative attitudes toward interpersonal communication in the workplace (Pradhan et al., 2020). Hostile atmosphere created by abusive supervision will weaken individuals’ appraisal of social intercourse, jeopardize their confidence in establishing and maintaining interpersonal interactions, and damage normal interpersonal relationships (Huang et al., 2019). Failing to meet the internal psychological needs to be respected and recognized by others, individuals will perceive a decline in their value and presence in the organization. Furthermore, this perception will raise doubts about the membership and lower their emotional identification (Ambrose and Ganegoda, 2020). Therefore, they are more likely to perceive marginalization (Dirican and Erdil, 2020).

Employees attach importance to how they are treated by the organization. A positive evaluation of themselves will be formed with preferential treatment (Bar-Kalifa and Atzil-Slonim, 2020). However, evaluation process of abusive supervision will lead to employees’ perception of being treated improperly and cognition of interpersonal strain in the workplace (Wang et al., 2020). Perception of the declining dignity and status due to abusive supervision (Burton and Barber, 2019) will dampen employees’ social interaction and bring social dilemmas in the workplace, leading to negative cognitions of self-esteem and self-concept in subsequent communication, then weaken emotional connection with the organization and generate workplace ostracism.

#### Workplace Ostracism and Employees’ Work Procrastination Behavior

With continuous perception of pressure situations and possible harm, employees’ emotional cognition and mental state will be negatively affected (Lisanne et al., 2019), and cause behavioral changes. Negative cognition will destroy the emotional bond of organizational identity (Howard et al., 2019) and reduce employees’ recognition with the organization. Thereby they are discouraged to contribute to the organization. Workplace ostracism breaks interpersonal communication and emotional recognition from employees’ perspective, threatens their basic emotion and dependency needs, easily inducing great psychological burden. To indirectly vent the painful emotions and dissatisfaction, they are more inclined to adopt defense mechanism to resist adverse emotional interference, then exhibit invisible negative feedback behaviors, such as lower work efficiency or organizational performance.

When employees generate workplace ostracism, they will not only experience strong negative emotions, but also become unable to effectively implement work tasks associated with organizational goals. Through individuals’ cognitive feedback on situational pressure, more negative evaluations will lead to resource depletion, such as psychological trauma and the weakening of self-control (Zhao et al., 2020). Hence employees will need extra time and energy to adjust themselves. These extra costs make them even more difficult to focus on the tasks (Aijing et al., 2019), and casue series of abnormal reactions. Workplace ostracism undermines employees’ sense of organizational justice and trust (Howard et al., 2019). Consequently, they will experience a continuing sense of powerlessness and fatigue over job security and career development (Wu et al., 2018). This negative sense will weaken organizational commitment, then become more likely to reduce work execution or even delay work tasks to cope with resource depletion (Mao et al., 2018).

#### The Mediating Effect of Workplace Ostracism

Abusive supervision is an interpersonal conflict without physical contact (Tepper et al., 2006), resulting in complex cognitive and emotional processes within the work environment. Leaders often direct unfriendly words or behaviors toward employees in the workplace. As a result, employees’ self-esteem and self-confidence will be damaged, with a strong sense of frustration and powerlessness. In this situation, employees tend to lower their self-appraisals and fall into social dilemmas (Wang et al., 2019). As posited by cognitive appraisal theory, negative cognitive evaluation will trigger fluctuations in psychological security and negatively impact behavioral responses (Majeed and Naseer, 2019). When employees are confronted by threatening event, continuing detrimental experience will cause negative psychological feelings or emotions (Peltokorpi and Ramaswami, 2019). With their intrinsic motivation and work willingness weakened, employees become more likely to adjust their behaviors to resist or avoid.

Information conveyed by leaders’ malicious treatment undermines the emotional channel between employees and the organization. Employees will generate workplace ostracism due to their sensitive cognition of the interpersonal pressure caused by occupational barriers, such as social alienation (De Clercq et al., 2019). Employees feel emotionally lost and distracted by the difficulty to feel emotional care and support from the organization (Anjum et al., 2019). This leads to the loss of work enthusiasm to act autonomously and a shift in attitude away from pro-organizational behavior, and further initiates unnecessary delays. Based on above reasoning, we proposed:

Hypothesis 1: Workplace ostracism mediates the relationship between abusive supervision and employees’ work procrastination behavior.

### Psychological Resilience and Psychological Detachment

#### The Moderating Effect of Psychological Resilience

As a core element of stable psychological characteristics (Garmezy, 1991), psychological resilience refers to the ability to “bounce back” from difficult experiences or dangerous situations (Connor and Davidson, 2003). This ability can also alleviate the negative cognitive evaluation of dynamic situations and events (King et al., 2016). It has been defined as an excellent willpower which helps individuals to overcome adversity and stay focused on the meaning of life (Liang et al., 2019). It also has a universal positive effect on maintaining physical and mental health. Resilient individuals can withstand high-intensity destructive changes based on their inner rebound ability, take initiative to prepare for the difficulties and resist the adverse effects of risk factors, and quickly make constructive self-adjustments (Lazarus, 1984).

Psychological resilience endues individual’s positive adaptability to enable their quick mental state rebound to its previous dynamic balance (Kathleen and Janyce, 2004). Therefore, they can minimize the negative impact of adversity or stress after suffering from crisis events. Individuals with high psychological resilience will apply positive emotional regulation strategies to buffer the cognitive bias induced by malignant experiences, reduce their negative evaluation of interpersonal interaction and generate optimistic thoughts (Crane and Searle, 2016). Cognition of identity within the organizational environment will be achieved. However, individuals with low psychological resilience are more likely to present immature psychological adaptation mechanism (Dray et al., 2014), leaving them poorly equipped to manage the perception of interpersonal dilemma in the workplace.

Abusive supervision leads to difficulties for employees to receive sufficient support and affirmation from their leaders (Agarwal, 2019). Their organizational identity and recognition by others from employees’ perspective will be damaged. Psychological resilience plays a protective role when individuals encounter abusive supervision, and helps them to adjust their psychological state to maintain mental health and restrain the adverse consequences. Therefore, the cognition of the damage of workplace dignity and the decline of workplace status shall be mitigated. Conversely, if employees lack relative stability in the face of stressful events, it will be difficult to conduct emotional adjustment and cognitive reconstruction when they encounter abusive supervision. Hence employees are more inclined to generate negative cognition interpretations of interpersonal relationships and social networks, such as workplace ostracism. In summary, we proposed:

Hypothesis 2: Psychological resilience moderates the relationship between abusive supervision and workplace ostracism.

#### The Moderating Effect of Psychological Detachment

Psychological detachment refers to the ability to mentally separate the areas of work and family (Park et al., 2011). Specifically, it means not only physically staying away from work environment during non-working hours, but also ceasing to think about and deal with work content, thereby consciously realizing the mental rest state of psychological liberation. As an important psychological defense method, psychological detachment effectively helps employees to divert attention (Chummar et al., 2019) and prevent them from being overcame by negative perception of workplace events. It also greatly diminishes the negative emotions caused by work-related interference and protects employees from resource damage. By contrast, employees who are unable to cut themselves off from work during breaks will devote even more resources to work-related matters, and their physical and mental systems will continue to be activated (Boekhorst et al., 2017). Inevitably their stress intensity and emotional exhaustion will increase (Chawla et al., 2020).

As a key way to experience restoration (Kilroy et al., 2020), psychological detachment enables emotional regulation to help individuals to restore working capital consumed by emotional labor and supplement mental energy. The repression and anxiety caused by work situations will consequently be eliminated or decreased and thus employees have sufficient energy and the best emotional state to invest in subsequent work (Ghosh and Sekiguchi, 2020). Conversely, without the process of mental recovery, employees in non-optimal state have to spend more time and energy to alleviate emotional injuries and recover lost resources. They will be unable to restore the basic state or reduce stress, and have to take extra efforts to resolve work issues and meet work needs. Be encountered with other severe workload, they won’t have sufficient working resources and mental state to embrace the challenge (Loveday et al., 2018), nor can they make appropriate behavioral decisions.

Workplace ostracism reduces employees’ expectation of social interaction. The reduction causes rapid disappearance of social resources and support networks (Kwan et al., 2018). They will be unable to restore emotional resources through effective social activities. However, relief from work roles and proper relaxation can relieve the tension and painful perception suffered from work, generate new resources and restore individuals’ function (Fritz and Sonnentag, 2006) to focus on work tasks. But employees with low psychological detachment cannot adjust their feelings through positive accumulation of recovery experiences, which will trigger physical and mental exhaustion (Chen and Li, 2018), and probably result in negative work attitude and failure to complete task within allotted time. Based on above discussion, we proposed:

Hypothesis 3: Psychological detachment moderates the relationship between workplace ostracism and employees’ work procrastination behavior.

## Methodology and Variable Design

### Survey Sample and Data Collection

Questionnaires were mainly distributed in Beijing, Shanghai, Tianjin, and Chongqing in China. As the questions are sensitive, we conducted surveys in organizations we had personal relationships, and HR managers recommended employees to participate in our research. Participants were selected from full-time employees of 32 enterprises in manufacturing, service, and education industries. With the diversity of sample sources, these samples presented wide coverage and well-representative. To avoid regional cultural bias and ensure validity of the questionnaire, a preliminary in-depth interview was conducted with five employees separately before formal implementation. It was done to guarantee participants fully understand the questions. Based on the feedback from these interviews, the questionnaire was improved to ensure surface validity and content validity, and thus reliability and rationality of the evaluation results were secured.

We contacted HR managers and obtained their consent first, and provided training and explanation to employees on how to fill the questionnaires. We also clarified that the surveys were anonymous and would be only used for academic research. At formal investigation stage, data was collected through time-lagged surveys in three stages over four months, and a total of 400 questionnaires were distributed. First survey required employees to report their demographic information. They were also instructed to complete items on abusive supervision, psychological resilience, and psychological disengagement, in order to arouse their awareness of abusive supervision. Two months later, they were asked to self-evaluate the feelings of workplace ostracism to observe the impact of abusive supervision on their psychological cognition. After another two months, they were asked to self-evaluate their work procrastination behavior to observe behavioral changes caused by cognition. Two-time intervals of two months were conducted in this research, not only to ensure more interactions between employees and the organization, but also to assess cognition and behavior changes. After eliminating 22 invalid questionnaires, 378 valid retained questionnaires presented an effective response rate of 94.50%. Among these samples, males accounted for 46.83%; 30 years old and below accounted for 43.92%, 31–50 years old for 52.65%, and over 51 years old for 3.44%, indicating a large majority was young or middle-aged; Below undergraduate, undergraduate, and above undergraduate accounted for 33.07, 32.54, and 34.39% respectively, indicating a large majority was knowledge worker; Tenure of 5 years and below accounted for 48.15%, 6–10 years for 24.60%, 11 years and above for 27.25%; Ordinary employees and low-level, mid-level, senior executives respectively accounted for 70.11, 20.90, 5.82, and 3.17%.

### Questionnaire Design and Variable Measurement

Responses were given on five-point likert scale, with higher scores indicating higher values. To ensure reliability and validity of the measurement tools, we followed Brislin’s (1976) translation and back-translation method, then adjusted expressions according to the specific situation of the surveys. All following scales have been verified in empirical research of Chinese organizational context.

#### Abusive Supervision

We adapted the five-item abusive leadership scale compiled by Mitchell and Ambrose (2012). Sample statements include “My supervisor often ridicules me in front of others” and “My supervisor is rude to me.” These five items were measured using 5-point likert scale (1 = strongly disagree to 5 = strongly agree). In this research, Cronbach’s alpha reliability coefficient was 0.906, reaching psychometric criteria.

#### Workplace Ostracism

We used Zhao and Liu’s (2019) five-item workplace ostracism scale, which they adapted from Ferris et al.’ (2008) research. Sample statements include “Some colleagues are not willing to work with me” and “Some colleagues refused to talk to me at work”. These five items were measured using 5-point likert scale (1 = strongly disagree to 5 = strongly agree). In this research, Cronbach’s alpha reliability coefficient was 0.910.

#### Work Procrastination Behavior

Following recommendations of Chu and Choi (2005), we used five items from Harriott and Ferrari’s (1996) six-item procrastination scale. Sample statements include “I usually postpone start of the task” and “I will postpone work that is not necessary at the moment.” These six items were measured using 5-point likert scale (1 = strongly disagree to 5 = strongly agree). In this research, Cronbach’s alpha reliability coefficient was 0.949.

#### Psychological Resilience

We adapted the six-item psychological resilience scale compiled by Luthans et al. (2007). Sample statements include “I can easily recover from frustration then continue to work” and “I usually take pressure at work calmly.” These six items were measured using 5-point likert scale (1 = strongly disagree to 5 = strongly agree). In this research, Cronbach’s alpha reliability coefficient was 0.984.

#### Psychological Detachment

We adapted the four-item recovery experience scale developed by Sonnentag and Fritz (2015), which is the most commonly used psychological detachment measurement tool. Sample statements include “I can leave work-related matters during non-work hours” and “I can get break from the demands of work.” These four items were measured using 5-point likert scale (1 = strongly disagree to 5 = strongly agree). In this research, Cronbach’s alpha reliability coefficient was 0.925.

#### Control Variables

We adopted conventional practice of controlling participants’ demographic characteristics, specifically gender, age, education, tenure, and position (Mazzola and Disselhorst, 2019).

## Results

### Common Method Bias Test

Research process was controlled in terms of anonymous responses and standardized question-answering procedures. However, as all data was self-reported by employees, their subjective feelings may result in artificial co-variation among variables. Therefore, it is necessary to test common method bias. Harman’s single-factor test generated five factors with the eigenvalue greater than 1, which is consistent with the number of variables in this research. Cumulative interpretation variance of these factors was 81.153%, and the maximum variance contribution of common factor was 31.397%. There was no single factor explaining most of the variation. Thus, there is no significant common method bias in research data.

### Confirmatory Factor Analysis

Confirmatory factor analysis was performed in AMOS to test structural validity of the model. Table 1 reports the results. Compared with other models, five-factor model shows the best fit (χ^2^ = 1189.676; df = 265; χ^2^/df = 4.489; RMSEA = 0.069; NFI = 0.905; IFI = 0.925; TLI = 0.914; CFI = 0.924), indicating that these five variables have good discriminant validity and belong to different constructs. In addition, factor loading of every item reached ideal standard of 0.60, and there is statistical significance among the items, indicating that these five variables have good convergent validity.

**TABLE 1:** Confirmatory factor analysis.

Model	Variables	χ^2^	df	χ^2^/df	RMSEA	NFI	IFI	TLI	CFI
**1**	AS; WO; WPB; PR; PD	1189.676	265	4.489	0.069	0.905	0.925	0.914	0.924
**2**	AS; WO; WPB; PR + PD	1870.516	269	6.954	0.126	0.851	0.869	0.854	0.869
**3**	AS + PR; WO + PD; WPB	2704.536	272	9.943	0.154	0.784	0.801	0.780	0.801
**4**	AS + WO + PR + PD; WPB	3389.851	274	12.372	0.174	0.729	0.746	0.721	0.745
**5**	AS + WO + WPB + PR + PD	4158.481	275	15.122	0.194	0.668	0.683	0.653	0.682

*AS, Abusive Supervision; WO, Workplace Ostracism; WPB, Work Procrastination Behavior; PR, Psychological Resilience; PD, Psychological Detachment.*

### Descriptive Statistics and Correlation Analysis

As shown in Table 2: Abusive supervision is significantly positively correlated with workplace ostracism and work procrastination behavior, while there are significant negative correlations between psychological resilience and workplace ostracism, as well as psychological detachment and work procrastination behavior. These results preliminarily verified theoretical hypothesis.

**TABLE 2:** Descriptive statistics and correlation analysis.

	Mean	SD	1	2	3	4	5	6	7	8	9	10
1 Gender	1.530	0.500	**–**									
2 Age	33.160	7.006	−0.107*	**–**								
3 Education	3.130	1.519	−0.034	−0.060	**–**							
4 Tenure	8.380	7.853	−0.167**	0.813***	−0.055	**–**						
5 Position	1.980	1.131	−0.069	0.301***	0.097	0.286***	**–**					
6 Abusive Supervision	3.022	1.099	−0.136**	0.027	0.065	0.047	0.092	**0.906**				
7 Workplace Ostracism	3.148	1.012	−0.123*	0.029	0.088	0.074	0.092	0.664***	**0.910**			
8 Work Procrastination Behavior	3.440	1.116	−0.116*	0.053	0.143**	0.163**	−0.041	0.476***	0.688***	**0.949**		
9 Psychological Resilience	2.568	1.369	0.068	0.004	−0.116*	−0.115*	0.051	−0.544***	−0.730***	−0.741***	**0.984**	
10 Psychological Detachment	2.643	1.176	0.130*	−0.020	−0.154**	−0.134**	0.077	−0.531***	−0.722***	−0.890***	0.808***	**0.925**

Numbers on diagonal (bold) are Cronbach’s alpha reliability coefficients. *p < 0.05. **p < 0.01. ***p < 0.001.

### Hypothesis Testing

We used SPSS and Process macro to test theoretical hypothesis. Indirect effect of abusive supervision on employees’ work procrastination behavior is 0.4289, 95%CI = [0.3399, 0.5291], reaching significant level. Therefore, Hypothesis 1 is supported.

Data analysis results of model 4 in Table 3 showed that interaction term between abusive supervision and psychological resilience has a significant negative impact on workplace ostracism (β = −0.075, *p* < 0.05), which is inconsistent with the direction of the effect of abusive supervision on workplace ostracism (β = 0.602, *p* < 0.001). Therefore, with the moderating effect of psychological resilience, the effect of abusive supervision on workplace ostracism is weakened (β = 0.315, *p* < 0.001). Hypothesis 2 is supported.

**TABLE 3:** Moderating effect of psychological resilience.

	Workplace Ostracism			
	Model 1	Model 2	Model 3	Model 4
Gender	−0.215*	−0.048	−0.082	−0.094
Age	−0.014	−0.010	0.009	0.007
Education	0.052	0.029	−0.007	−0.010
Tenure	0.015	0.012	−0.011	−0.010
Position	0.064	0.018	0.083**	0.078**
Abusive Supervision		0.602***	0.325***	0.315***
Psychological Resilience			−0.408***	−0.435***
Abusive Supervision × Psychological Resilience				−0.075*
*F*	2.506*	50.062***	95.688***	85.300***
*R*^2^	0.033	0.447	0.644	0.649
Adjust *R*^2^	0.020	0.438	0.637	0.641
*R*^2^ Change	0.033	0.415	0.197	0.005

* p < 0.05, ** p < 0.01, *** p < 0.001.

**FIGURE 2:**
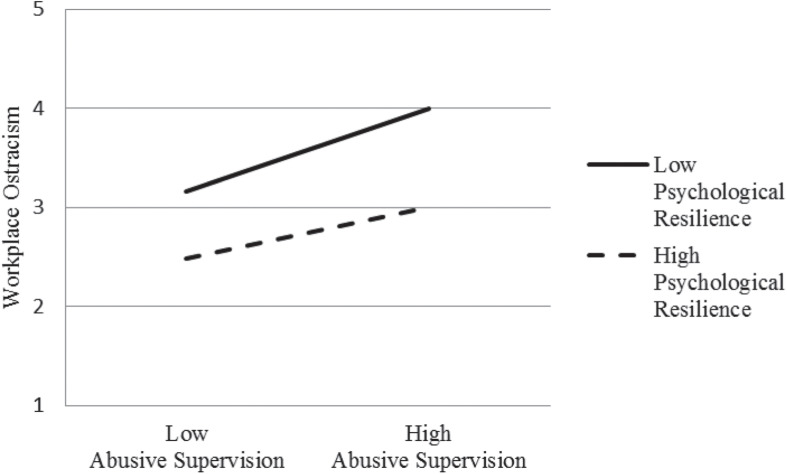
Interaction effect of psychological resilience.

Figure 2 further illustrated that psychological resilience plays an interfering moderating effect in the process of abusive supervision positively affecting workplace ostracism. Under the condition of low psychological resilience, regression slope of abusive supervision positively affecting workplace ostracism is relatively inclined; Under the condition of high psychological resilience, regression slope is relatively flat and low.

According to data analysis results of model 4 in Table 4, interaction term between workplace ostracism and psychological detachment can significantly negatively affect employee’s work procrastination behavior (β = −0.040, *p* < 0.05), which is inconsistent with the direction of workplace ostracism affecting employee’s work procrastination behavior (β = 0.745, *p* < 0.001). Therefore, with the moderating effect of psychological detachment, the effect of workplace ostracism on employee’s work procrastination behavior is weakened (β = 0.104, *p* < 0.01). Hypothesis 3 is supported.

**TABLE 4:** Moderating effect of psychological detachment.

	Work procrastination behavior			
	Model 1	Model 2	Model 3	Model 4
Gender	−0.180	−0.020	0.024	0.026
Age	−0.032*	−0.022*	−0.002	0.000
Education	0.116**	0.077**	0.009	0.008
Tenure	0.050***	0.038***	0.009	0.007
Position	−0.100	−0.148***	−0.001	−0.001
Workplace Ostracism		0.745***	0.109**	0.104**
Psychological Detachment			−0.769***	−0.760***
Workplace Ostracism × Psychological Detachment				−0.040*
*F*	6.663***	67.911***	210.357***	184.970***
*R*^2^	0.082	0.523	0.799	0.800
Adjust *R*^2^	0.070	0.516	0.795	0.796
*R*^2^ Change	0.082	0.441	0.276	0.002

*p < 0.05. **p < 0.01. ***p < 0.001.

**FIGURE 3:**
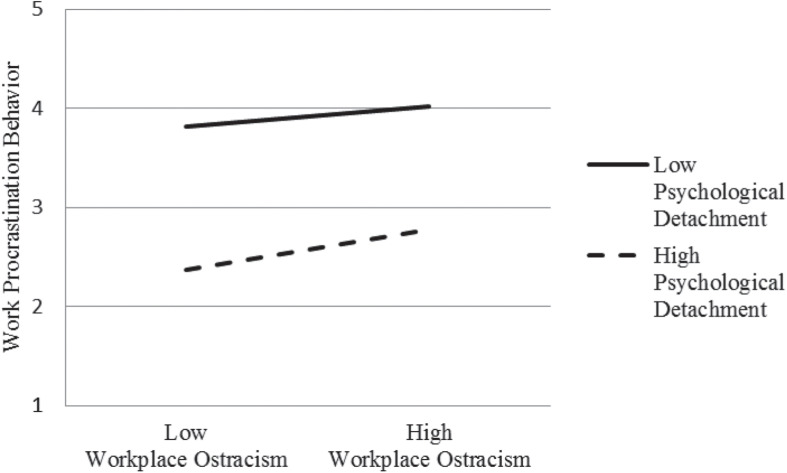
Interaction effect of psychological detachment.

As can be seen in Figure 3, diagram of the interactive effect of psychological detachment is consistent with hypothesis 3: When the degree of psychological detachment is low, work procrastination behavior appears to be higher; When the degree of psychological detachment is high, work procrastination behavior appears to be lower.

## Discussion

### Theoretical Significance

First, based on cognitive appraisal theory, this research explained the occurrence of work procrastination behavior as a consequence of abusive supervision, and further expanded the antecedents of work procrastination behavior from the perspective of employees’ passive choice. Previous researches have confirmed that employees will experience persistent negative feelings when they are subjected to abusive supervision. This feeling is considered as an emotionally response to workplace stress. After employees cognizing the demeaning and humiliating information transmitted by abusive supervision, they will perceive that they are treated unfairly within the organization. In response, their organizational commitment and job performance will decline, then become more inclined to engage in work procrastination behavior. Different from the perspective of employees’ active choice in previous research, we conceptualized work procrastination behavior as employees’ passive behavioral response after encountering negative situation (Pan et al., 2018). It is pointed out that employees’ passive acceptance of negative management behaviors will also affect their subjective feelings, and induce work procrastination behavior. It’s not aiming at restoring emotional resources or diverting attention (Ainslie, 1975; Folkman, 1984; O’Donoghue and Rabin, 1999), but presenting powerlessness. Therefore, work procrastination behavior is a behavior result of employees’ maladjustment in face of abusive supervision.

Second, we analyzed the impact of abusive supervision on employees’ work procrastination behavior through the intermediary perspective of workplace ostracism. Most research set workplace ostracism as an independent variable to explore its impact on counterproductive behavior. But our empirical results confirmed the impact of abusive supervision on workplace ostracism, and further revealed the formation of workplace ostracism. Low-quality leader-member relationships will lead to employees’ initiative to reduce interaction and exchanges with leaders. It makes them difficult to gain leaders’ trust, support, and affirmation, which are required by employees to meet their needs for recognition of capabilities and values within the organization (Huang et al., 2020). As a consequent positive self-evaluation cannot be established. Employees subjected to abusive supervision will be frustrated by the loss of self-esteem and organizational status. This frustration reduces the possibility of obtaining emotional support from interpersonal network, and employees may subjectively feel deliberate ostracism from others (Karagonlar and Neves, 2020) and further suffer emotional contusion. Above research findings provide a more in-depth explanation that employees’ psychological distress caused by abusive supervision will induce cognitive reaction of workplace ostracism.

Third, based on the mediating effect of workplace ostracism, we provided a more comprehensive theoretical explanation of psychological resilience moderating cognitive process and psychological detachment moderating behavioral process in the same model of the formation mechanism of work procrastination behavior. Psychological resilience endows employees’ optimistic attitude and sufficient confidence to deal with stressful events, so they can mobilize protective psychological potential to identify stressors as temporary challenge (Crane and Searle, 2016). Consequently, they are more likely to perceive information in a constructive way and create a conducive cognitive environment in the risk. However, employees with low psychological resilience lack identity with reality, and thus they are more sensitive to interpersonal conflict and likely to generate workplace ostracism. On the other hand, the moderating effect of psychological detachment further explained Yang et al.’s (2017) opinion that “restoration experience is significantly correlated with subsequent post performance.” If employees have difficulty in staying away from work environment to get recovery from the hurt by workplace ostracism, continuous consumption of limited resources will weaken their self-regulation and control ability. This possibility of depressed and self-frustrated of employees in the workplace will increase, just as their inclination to engage in work procrastination behavior. However, diagram of the interaction effect of psychological detachment also shows that under the condition of high psychological detachment, regression slope of workplace ostracism affecting work procrastination behavior is higher than it under the condition of low psychological detachment. Excessive psychological detachment may better explains employees’ difficulties to return back to work environment after relieving their physical and mental state away from workplace. More procrastination behaviors will take place at work as a result.

### Practical Significance

First, we attempted to explore the effect of abusive supervision on employees’ work procrastination behavior, providing theoretical and practical supports for leaders on how to avoid the destructive effects of abusive supervision and effectively manage employees, and exerting a “precautionary” function. Especially during epidemic, remote working increases frequency of employees’ work procrastination behavior and its probability of occurrence. Understanding antecedents of work procrastination behavior will be crucial for all prevention actions taking up in organizations. Abusive supervision inevitably has a negative impact on employees (Peltokorpi and Ramaswami, 2019). They may delay the process of work affairs in order to release negative cognition. Therefore, leaders should pay attention to the establishment of their own behavioral norms, provide effective supervision to employees, and set up formal organizational platforms or reliable channels to convey employees’ demands or complaints. In addition, leaders’ interpersonal care should be promoted in the workplace in return for employee’s gratitude and trust. By doing that, their pro-organization motivation, dedication and willingness to perform their duties will be enhanced. Therefore, it is necessary for leaders to focus on the promotion of interaction and trust with employees, strive to establish a better leader-member exchange relationship, in order to encourage the diversity and tolerance in the workplace, and development a culture atmosphere of respect among employees.

Second, workplace ostracism stems from individuals’ subjective perception of organizational environment. Remote working under epidemic situation brings more distance between organizational members. With fewer real contact and emotional communication, workplace ostracism seems to be more frequent. But the support and help from other organizational members highlight humanized organizational atmosphere. Moreover, stable interpersonal relationships will enhance employees’ emotional identity and job satisfaction, and increase their sense of self-identity by improving organization-based self-esteem. Higher work confidence can help employees to build reasonable social network within the organization and accumulate more social capital by strengthening links with other organizational members and repairing damaged relationships (Zhang et al., 2020). In addition, organizations should provide employees with more opportunities to participate in decision-making, so that employees will feel that they are an important part of the group or have a special organizational status. Therefore, they will be encouraged to accept more challenging tasks and realize self-worth. When employees regard themselves as an important organizational member, they will have a sense of belonging to the organization. This sense of belonging is critical for them to maintain positive cognition and improving individuals’ happiness, and actively practice role behavior. Therefore, the possibility of employees’ pessimistic attitudes and cognitions can be reduced by shaping organizational culture of friendly interactions and cultivating optimistic awareness of belonging.

Third, individual trait factors influence the impact of abusive supervision on employees’ work procrastination behavior through workplace ostracism. Psychological resilience affects employees’ perceptions of organizational environment and helps them to reduce worries about challenges or difficulties, and thus employees think actively in adversity. Strong ability to manage psychological state can become a source of motivation for employees to overcome negative effects, and help them to face leaders’ abusive behaviors flexibly. Employees will be able to control negative cognition and conduct self-reexamination (Conway et al., 2019). Therefore, positive mentality and resilience ability are particularly important. Establishment of highly caring and training-oriented organizational culture can create a good environment for employees to improve their psychological adaptability and coping capabilities. In addition, helping employees to restore psychological resources will temper their impulse to procrastinate work tasks. By improving employees’ welfare and remuneration, we can provide them with sufficient social supports without ignoring their demands. Ultimately, they are encouraged to form a positive attitude toward the organization. On the other hand, organizations should consider to arrange work tasks legitimately with appropriate flexible time, and help employees to adjust to the best working condition. Employees should also learn to take appropriate breaks during non-working hours and proactive measures to avoid excessively interreference of work affairs over their family life.

### Research Limitations and Future Directions

First, variables used in this research were measured by employees’ subjective evaluation. Particularity and sensitivity of the variables may result in the deviation of respondents’ answers from facts, as they may tend to protect personal privacy and engage in impression management. As a result, the effect values cannot be accurately calculated or tested during research process. Then raised questions about response bias between questionnaire evaluation results and actual situation, as well as reliability of research conclusions. Future research can use experimental methods or collect pairing data to further verify the robustness of research results. In addition, we used time-lagged method to obtain data. It reduces common method bias to a certain extent and reflects a connotation of time change, but cannot effectively infer causal relationship between variables. So, longitudinal measurement method should be used to effectively examine their causality in future.

Second, we included workplace ostracism into the research of abusive supervision affecting employees’ work procrastination behavior. Initially the mediating effect of workplace ostracism was confirmed. Also, it means that there may be other variables mediate the relationship between these two variables. So, further researches need to explore other potential mediating variables to better reveal the relationship. In addition, we didn’t explore antecedent variables of abusive supervision in model. Currently, some scholars have already discussed these antecedent variables of abusive supervision, and verified some key mechanisms. Future researches should extend the model to investigate how antecedent variables of abusive supervision potentially and chronically affect employees’ work procrastination behavior through different causal mechanisms. On the other hand, we only considered the controlling effect of demographic information and the moderating effect of personal characteristics at individual level. Future research should further improve the model by introducing factors at organizational level into model, such as organizational performance orientation, organizational political atmosphere, and organizational cultural environment. It is also required to explore the existence of difference in mechanism or strength among different types of organizations.

Third, we explored the mechanism of abusive supervision influencing employees’ work procrastination behavior in Chinese organizational context, and it is conducive to perfecting localized leadership theory. However, path between abusive supervision and employees’ work procrastination behavior implies the meaning of high-power distance in Chinese organizations. In order to avoid leaders’ further retaliation and punishment, employees don’t dare to impose hostility or conflict behaviors on leaders directly (Harris et al., 2007), so they can only respond to leaders’ non-physical violence with depressed work attitude and hidden retreat. The mediating effect of workplace ostracism also shows collectivist orientation and leader-centered principles in Chinese organizational context. Therefore, it is unclear whether theoretical model proposed in this research is valid in different cultures. Strength of the effect or the mechanism has not been confirmed. Future research can expand breadth of this research and explore the influence of different international environments on the relationships between these variables.

## Conclusion

We further elaborated antecedent variables of employees’ work procrastination behavior. Abusive supervision has a significant positive impact on employees’ work procrastination behavior through the mediating effect of workplace ostracism. This process is also moderated by psychological resilience and psychological detachment. These findings are attributable to such employees often lack ability to rebound or recover when facing setbacks and pressures. Therefore, organizations consciously reduce or prevent employees from engaging in work procrastination behavior, not only require leaders to optimize their management methods and leadership styles, but also require employees to control psychological cognition and improve psychological literacy.

## Data Availability Statement

The raw data supporting the conclusions of this article will be made available by the authors, without undue reservation.

## Ethics Statement

This research involving human participants was reviewed and approved by the Ethical Review Committee of Jiangsu University in China. The patients/participants provided their written informed consent to participate in this research.

## Author Contributions

All authors listed have made a substantial, direct and intellectual contribution to the work, and approved it for publication.

## Conflict of Interest

The authors declare that the research was conducted in the absence of any commercial or financial relationships that could be construed as a potential conflict of interest.
